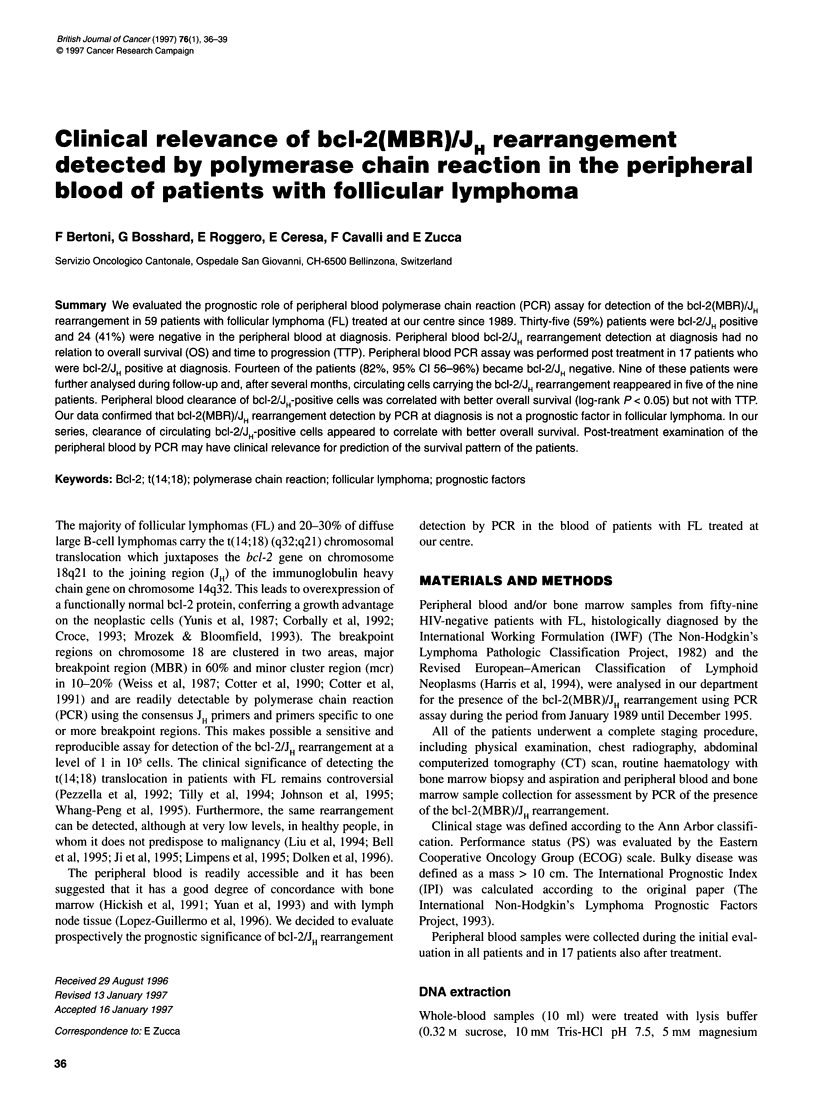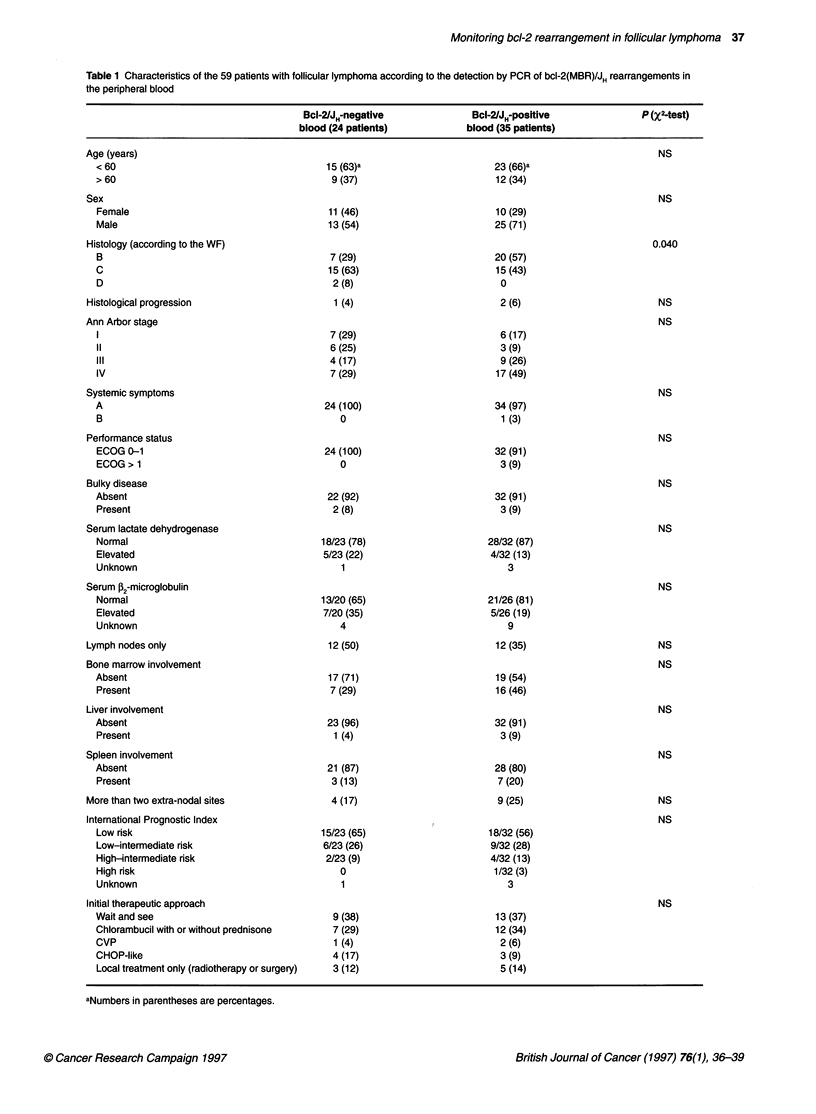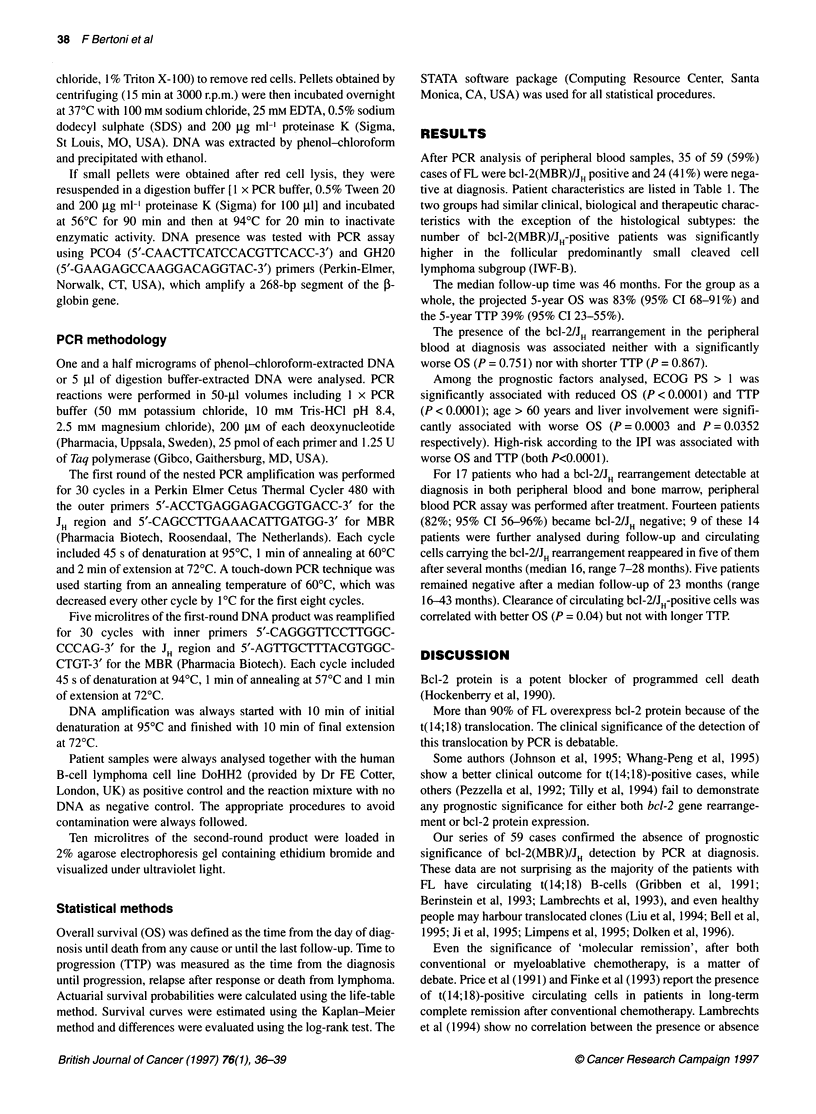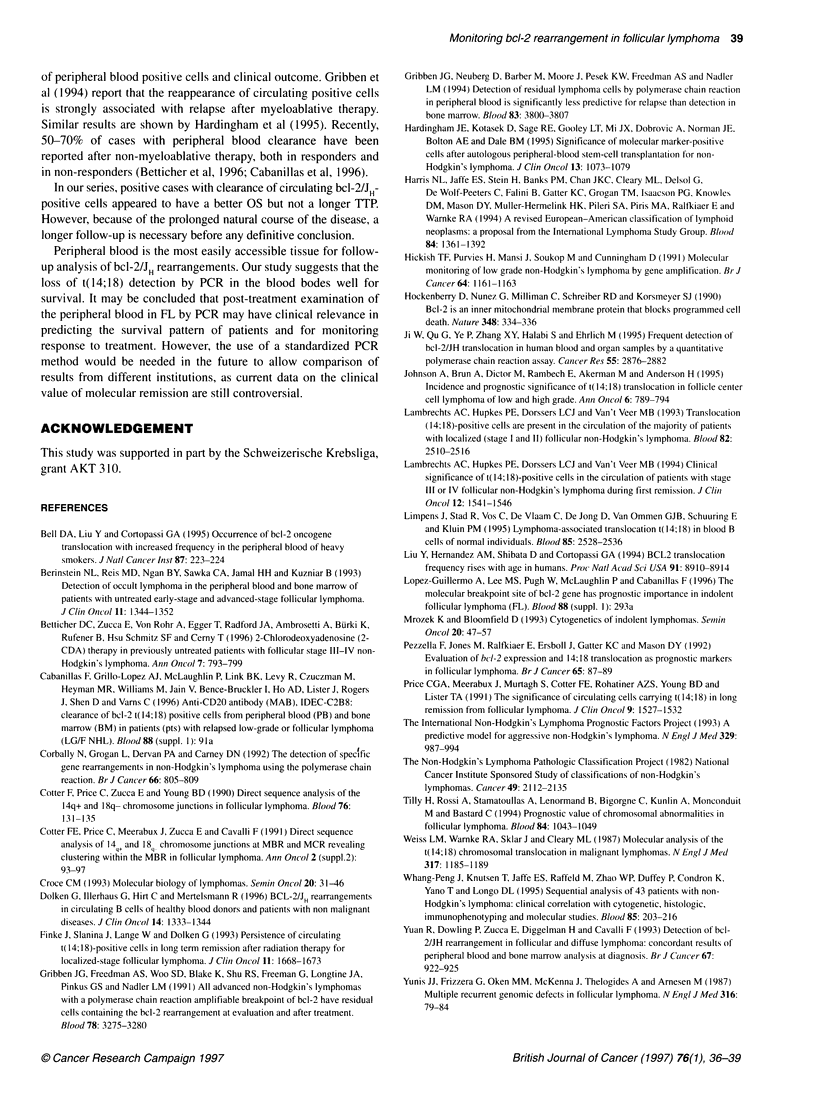# Clinical relevance of bcl-2(MBR)/J(H) rearrangement detected by polymerase chain reaction in the peripheral blood of patients with follicular lymphoma.

**DOI:** 10.1038/bjc.1997.332

**Published:** 1997

**Authors:** F. Bertoni, G. Bosshard, E. Roggero, E. Ceresa, F. Cavalli, E. Zucca

**Affiliations:** Servizio Oncologico Cantonale, Ospedale San Giovanni, Bellinzona, Switzerland.

## Abstract

We evaluated the prognostic role of peripheral blood polymerase chain reaction (PCR) assay for detection of the bcl-2(MBR)/J(H) rearrangement in 59 patients with follicular lymphoma (FL) treated at our centre since 1989. Thirty-five (59%) patients were bcl-2/J(H) positive and 24 (41%) were negative in the peripheral blood at diagnosis. Peripheral blood bcl-2/J(H) rearrangement detection at diagnosis had no relation to overall survival (OS) and time to progression (TTP). Peripheral blood PCR assay was performed post treatment in 17 patients who were bcl-2/J(H) positive at diagnosis. Fourteen of the patients (82%, 95% CI 56-96%) became bcl-2/J(H) negative. Nine of these patients were further analysed during follow-up and, after several months, circulating cells carrying the bcl-2/J(H) rearrangement reappeared in five of the nine patients. Peripheral blood clearance of bcl-2/J(H)-positive cells was correlated with better overall survival (log-rank P < 0.05) but not with TTP. Our data confirmed that bcl-2(MBR)/J(H) rearrangement detection by PCR at diagnosis is not a prognostic factor in follicular lymphoma. In our series, clearance of circulating bcl-2/J(H)-positive cells appeared to correlate with better overall survival. Post-treatment examination of the peripheral blood by PCR may have clinical relevance for prediction of the survival pattern of the patients.


British Journal of Cancer (1997) 76(1), 36–39
© 1997 Cancer Research Campaign

### Clinical relevance of bcl-2(MBR)/$J_H$ rearrangement detected by polymerase chain reaction in the peripheral blood of patients with follicular lymphoma

**F Bertoni, G Bosshard, E Roggero, E Ceresa, F Cavalli and E Zucca**

Servizio Oncologico Cantonale, Ospedale San Giovanni, CH-6500 Bellinzona, Switzerland

**Summary** We evaluated the prognostic role of peripheral blood polymerase chain reaction (PCR) assay for detection of the bcl-2(MBR)/$J_H$ rearrangement in 59 patients with follicular lymphoma (FL) treated at our centre since 1989. Thirty-five (59%) patients were bcl-2/$J_H$ positive and 24 (41%) were negative in the peripheral blood at diagnosis. Peripheral blood bcl-2/$J_H$ rearrangement detection at diagnosis had no relation to overall survival (OS) and time to progression (TTP). Peripheral blood PCR assay was performed post treatment in 17 patients who were bcl-2/$J_H$ positive at diagnosis. Fourteen of the patients (82%, 95% CI 56–96%) became bcl-2/$J_H$ negative. Nine of these patients were further analysed during follow-up and, after several months, circulating cells carrying the bcl-2/$J_H$ rearrangement reappeared in five of the nine patients. Peripheral blood clearance of bcl-2/$J_H$-positive cells was correlated with better overall survival (log-rank $P < 0.05$) but not with TTP. Our data confirmed that bcl-2(MBR)/$J_H$ rearrangement detection by PCR at diagnosis is not a prognostic factor in follicular lymphoma. In our series, clearance of circulating bcl-2/$J_H$-positive cells appeared to correlate with better overall survival. Post-treatment examination of the peripheral blood by PCR may have clinical relevance for prediction of the survival pattern of the patients.

**Keywords:** Bcl-2; t(14;18); polymerase chain reaction; follicular lymphoma; prognostic factors

The majority of follicular lymphomas (FL) and 20–30% of diffuse large B-cell lymphomas carry the t(14;18) (q32;q21) chromosomal translocation which juxtaposes the *bcl-2* gene on chromosome 18q21 to the joining region ($J_H$) of the immunoglobulin heavy chain gene on chromosome 14q32. This leads to overexpression of a functionally normal bcl-2 protein, conferring a growth advantage on the neoplastic cells (Yunis et al, 1987; Corbally et al, 1992; Croce, 1993; Mrozek & Bloomfield, 1993). The breakpoint regions on chromosome 18 are clustered in two areas, major breakpoint region (MBR) in 60% and minor cluster region (mcr) in 10–20% (Weiss et al, 1987; Cotter et al, 1990; Cotter et al, 1991) and are readily detectable by polymerase chain reaction (PCR) using the consensus $J_H$ primers and primers specific to one or more breakpoint regions. This makes possible a sensitive and reproducible assay for detection of the bcl-2/$J_H$ rearrangement at a level of 1 in $10^5$ cells. The clinical significance of detecting the t(14;18) translocation in patients with FL remains controversial (Pezzella et al, 1992; Tilly et al, 1994; Johnson et al, 1995; Whang-Peng et al, 1995). Furthermore, the same rearrangement can be detected, although at very low levels, in healthy people, in whom it does not predispose to malignancy (Liu et al, 1994; Bell et al, 1995; Ji et al, 1995; Limpens et al, 1995; Dolken et al, 1996).

The peripheral blood is readily accessible and it has been suggested that it has a good degree of concordance with bone marrow (Hickish et al, 1991; Yuan et al, 1993) and with lymph node tissue (Lopez-Guillermo et al, 1996). We decided to evaluate prospectively the prognostic significance of bcl-2/$J_H$ rearrangement detection by PCR in the blood of patients with FL treated at our centre.

*Received 29 August 1996*
*Revised 13 January 1997*
*Accepted 16 January 1997*

*Correspondence to:* E Zucca

### MATERIALS AND METHODS

Peripheral blood and/or bone marrow samples from fifty-nine HIV-negative patients with FL, histologically diagnosed by the International Working Formulation (IWF) (The Non-Hodgkin's Lymphoma Pathologic Classification Project, 1982) and the Revised European–American Classification of Lymphoid Neoplasms (Harris et al, 1994), were analysed in our department for the presence of the bcl-2(MBR)/$J_H$ rearrangement using PCR assay during the period from January 1989 until December 1995.

All of the patients underwent a complete staging procedure, including physical examination, chest radiography, abdominal computerized tomography (CT) scan, routine haematology with bone marrow biopsy and aspiration and peripheral blood and bone marrow sample collection for assessment by PCR of the presence of the bcl-2(MBR)/$J_H$ rearrangement.

Clinical stage was defined according to the Ann Arbor classification. Performance status (PS) was evaluated by the Eastern Cooperative Oncology Group (ECOG) scale. Bulky disease was defined as a mass > 10 cm. The International Prognostic Index (IPI) was calculated according to the original paper (The International Non-Hodgkin's Lymphoma Prognostic Factors Project, 1993).

Peripheral blood samples were collected during the initial evaluation in all patients and in 17 patients also after treatment.

### DNA extraction

Whole-blood samples (10 ml) were treated with lysis buffer (0.32 M sucrose, 10 mM Tris-HCl pH 7.5, 5 mM magnesium

**Table 1** Characteristics of the 59 patients with follicular lymphoma according to the detection by PCR of bcl-2(MBR)/$J_H$ rearrangements in the peripheral blood

| | **Bcl-2/$J_H$-negative blood (24 patients)** | **Bcl-2/$J_H$-positive blood (35 patients)** | ***P* ($\chi^2$-test)** |
|---|---|---|---|
| Age (years) | | | NS |
| < 60 | 15 (63)[a] | 23 (66)[a] | |
| > 60 | 9 (37) | 12 (34) | |
| Sex | | | NS |
| Female | 11 (46) | 10 (29) | |
| Male | 13 (54) | 25 (71) | |
| Histology (according to the WF) | | | 0.040 |
| B | 7 (29) | 20 (57) | |
| C | 15 (63) | 15 (43) | |
| D | 2 (8) | 0 | |
| Histological progression | 1 (4) | 2 (6) | NS |
| Ann Arbor stage | | | NS |
| I | 7 (29) | 6 (17) | |
| II | 6 (25) | 3 (9) | |
| III | 4 (17) | 9 (26) | |
| IV | 7 (29) | 17 (49) | |
| Systemic symptoms | | | NS |
| A | 24 (100) | 34 (97) | |
| B | 0 | 1 (3) | |
| Performance status | | | NS |
| ECOG 0–1 | 24 (100) | 32 (91) | |
| ECOG > 1 | 0 | 3 (9) | |
| Bulky disease | | | NS |
| Absent | 22 (92) | 32 (91) | |
| Present | 2 (8) | 3 (9) | |
| Serum lactate dehydrogenase | | | NS |
| Normal | 18/23 (78) | 28/32 (87) | |
| Elevated | 5/23 (22) | 4/32 (13) | |
| Unknown | 1 | 3 | |
| Serum $\beta_2$-microglobulin | | | NS |
| Normal | 13/20 (65) | 21/26 (81) | |
| Elevated | 7/20 (35) | 5/26 (19) | |
| Unknown | 4 | 9 | |
| Lymph nodes only | 12 (50) | 12 (35) | NS |
| Bone marrow involvement | | | NS |
| Absent | 17 (71) | 19 (54) | |
| Present | 7 (29) | 16 (46) | |
| Liver involvement | | | NS |
| Absent | 23 (96) | 32 (91) | |
| Present | 1 (4) | 3 (9) | |
| Spleen involvement | | | NS |
| Absent | 21 (87) | 28 (80) | |
| Present | 3 (13) | 7 (20) | |
| More than two extra-nodal sites | 4 (17) | 9 (25) | NS |
| International Prognostic Index | | | NS |
| Low risk | 15/23 (65) | 18/32 (56) | |
| Low–intermediate risk | 6/23 (26) | 9/32 (28) | |
| High–intermediate risk | 2/23 (9) | 4/32 (13) | |
| High risk | 0 | 1/32 (3) | |
| Unknown | 1 | 3 | |
| Initial therapeutic approach | | | NS |
| Wait and see | 9 (38) | 13 (37) | |
| Chlorambucil with or without prednisone | 7 (29) | 12 (34) | |
| CVP | 1 (4) | 2 (6) | |
| CHOP-like | 4 (17) | 3 (9) | |
| Local treatment only (radiotherapy or surgery) | 3 (12) | 5 (14) | |

[a]Numbers in parentheses are percentages.

*© Cancer Research Campaign 1997*

chloride, 1% Triton X-100) to remove red cells. Pellets obtained by centrifuging (15 min at 3000 r.p.m.) were then incubated overnight at 37°C with 100 mM sodium chloride, 25 mM EDTA, 0.5% sodium dodecyl sulphate (SDS) and 200 μg $ml^{-1}$ proteinase K (Sigma, St Louis, MO, USA). DNA was extracted by phenol–chloroform and precipitated with ethanol.

If small pellets were obtained after red cell lysis, they were resuspended in a digestion buffer [1 × PCR buffer, 0.5% Tween 20 and 200 μg $ml^{-1}$ proteinase K (Sigma) for 100 μl] and incubated at 56°C for 90 min and then at 94°C for 20 min to inactivate enzymatic activity. DNA presence was tested with PCR assay using PCO4 (5′-CAACTTCATCCACGTTCACC-3′) and GH20 (5′-GAAGAGCCAAGGACAGGTAC-3′) primers (Perkin-Elmer, Norwalk, CT, USA), which amplify a 268-bp segment of the β-globin gene.

### PCR methodology

One and a half micrograms of phenol–chloroform-extracted DNA or 5 μl of digestion buffer-extracted DNA were analysed. PCR reactions were performed in 50-μl volumes including 1 × PCR buffer (50 mM potassium chloride, 10 mM Tris-HCl pH 8.4, 2.5 mM magnesium chloride), 200 μM of each deoxynucleotide (Pharmacia, Uppsala, Sweden), 25 pmol of each primer and 1.25 U of *Taq* polymerase (Gibco, Gaithersburg, MD, USA).

The first round of the nested PCR amplification was performed for 30 cycles in a Perkin Elmer Cetus Thermal Cycler 480 with the outer primers 5′-ACCTGAGGAGACGGTGACC-3′ for the $J_H$ region and 5′-CAGCCTTGAAACATTGATGG-3′ for MBR (Pharmacia Biotech, Roosendaal, The Netherlands). Each cycle included 45 s of denaturation at 95°C, 1 min of annealing at 60°C and 2 min of extension at 72°C. A touch-down PCR technique was used starting from an annealing temperature of 60°C, which was decreased every other cycle by 1°C for the first eight cycles.

Five microlitres of the first-round DNA product was reamplified for 30 cycles with inner primers 5′-CAGGGTTCCTTGGC-CCCAG-3′ for the $J_H$ region and 5′-AGTTGCTTTACGTGGC-CTGT-3′ for the MBR (Pharmacia Biotech). Each cycle included 45 s of denaturation at 94°C, 1 min of annealing at 57°C and 1 min of extension at 72°C.

DNA amplification was always started with 10 min of initial denaturation at 95°C and finished with 10 min of final extension at 72°C.

Patient samples were always analysed together with the human B-cell lymphoma cell line DoHH2 (provided by Dr FE Cotter, London, UK) as positive control and the reaction mixture with no DNA as negative control. The appropriate procedures to avoid contamination were always followed.

Ten microlitres of the second-round product were loaded in 2% agarose electrophoresis gel containing ethidium bromide and visualized under ultraviolet light.

### Statistical methods

Overall survival (OS) was defined as the time from the day of diagnosis until death from any cause or until the last follow-up. Time to progression (TTP) was measured as the time from the diagnosis until progression, relapse after response or death from lymphoma. Actuarial survival probabilities were calculated using the life-table method. Survival curves were estimated using the Kaplan–Meier method and differences were evaluated using the log-rank test. The STATA software package (Computing Resource Center, Santa Monica, CA, USA) was used for all statistical procedures.

### RESULTS

After PCR analysis of peripheral blood samples, 35 of 59 (59%) cases of FL were bcl-2(MBR)/$J_H$ positive and 24 (41%) were negative at diagnosis. Patient characteristics are listed in Table 1. The two groups had similar clinical, biological and therapeutic characteristics with the exception of the histological subtypes: the number of bcl-2(MBR)/$J_H$-positive patients was significantly higher in the follicular predominantly small cleaved cell lymphoma subgroup (IWF-B).

The median follow-up time was 46 months. For the group as a whole, the projected 5-year OS was 83% (95% CI 68–91%) and the 5-year TTP 39% (95% CI 23–55%).

The presence of the bcl-2/$J_H$ rearrangement in the peripheral blood at diagnosis was associated neither with a significantly worse OS ($P = 0.751$) nor with shorter TTP ($P = 0.867$).

Among the prognostic factors analysed, ECOG PS > 1 was significantly associated with reduced OS ($P < 0.0001$) and TTP ($P < 0.0001$); age > 60 years and liver involvement were significantly associated with worse OS ($P = 0.0003$ and $P = 0.0352$ respectively). High-risk according to the IPI was associated with worse OS and TTP (both $P<0.0001$).

For 17 patients who had a bcl-2/$J_H$ rearrangement detectable at diagnosis in both peripheral blood and bone marrow, peripheral blood PCR assay was performed after treatment. Fourteen patients (82%; 95% CI 56–96%) became bcl-2/$J_H$ negative; 9 of these 14 patients were further analysed during follow-up and circulating cells carrying the bcl-2/$J_H$ rearrangement reappeared in five of them after several months (median 16, range 7–28 months). Five patients remained negative after a median follow-up of 23 months (range 16–43 months). Clearance of circulating bcl-2/$J_H$-positive cells was correlated with better OS ($P = 0.04$) but not with longer TTP.

### DISCUSSION

Bcl-2 protein is a potent blocker of programmed cell death (Hockenberry et al, 1990).

More than 90% of FL overexpress bcl-2 protein because of the t(14;18) translocation. The clinical significance of the detection of this translocation by PCR is debatable.

Some authors (Johnson et al, 1995; Whang-Peng et al, 1995) show a better clinical outcome for t(14;18)-positive cases, while others (Pezzella et al, 1992; Tilly et al, 1994) fail to demonstrate any prognostic significance for either both *bcl-2* gene rearrangement or bcl-2 protein expression.

Our series of 59 cases confirmed the absence of prognostic significance of bcl-2(MBR)/$J_H$ detection by PCR at diagnosis. These data are not surprising as the majority of the patients with FL have circulating t(14;18) B-cells (Gribben et al, 1991; Berinstein et al, 1993; Lambrechts et al, 1993), and even healthy people may harbour translocated clones (Liu et al, 1994; Bell et al, 1995; Ji et al, 1995; Limpens et al, 1995; Dolken et al, 1996).

Even the significance of 'molecular remission', after both conventional or myeloablative chemotherapy, is a matter of debate. Price et al (1991) and Finke et al (1993) report the presence of t(14;18)-positive circulating cells in patients in long-term complete remission after conventional chemotherapy. Lambrechts et al (1994) show no correlation between the presence or absence

 © Cancer Research Campaign 1997

of peripheral blood positive cells and clinical outcome. Gribben et al (1994) report that the reappearance of circulating positive cells is strongly associated with relapse after myeloablative therapy. Similar results are shown by Hardingham et al (1995). Recently, 50–70% of cases with peripheral blood clearance have been reported after non-myeloablative therapy, both in responders and in non-responders (Betticher et al, 1996; Cabanillas et al, 1996).

In our series, positive cases with clearance of circulating bcl-2/$J_H$-positive cells appeared to have a better OS but not a longer TTP. However, because of the prolonged natural course of the disease, a longer follow-up is necessary before any definitive conclusion.

Peripheral blood is the most easily accessible tissue for follow-up analysis of bcl-2/$J_H$ rearrangements. Our study suggests that the loss of t(14;18) detection by PCR in the blood bodes well for survival. It may be concluded that post-treatment examination of the peripheral blood in FL by PCR may have clinical relevance in predicting the survival pattern of patients and for monitoring response to treatment. However, the use of a standardized PCR method would be needed in the future to allow comparison of results from different institutions, as current data on the clinical value of molecular remission are still controversial.

### ACKNOWLEDGEMENT

This study was supported in part by the Schweizerische Krebsliga, grant AKT 310.

### REFERENCES

Bell DA, Liu Y and Cortopassi GA (1995) Occurrence of bcl-2 oncogene translocation with increased frequency in the peripheral blood of heavy smokers. *J Natl Cancer Inst* **87**: 223–224

Berinstein NL, Reis MD, Ngan BY, Sawka CA, Jamal HH and Kuzniar B (1993) Detection of occult lymphoma in the peripheral blood and bone marrow of patients with untreated early-stage and advanced-stage follicular lymphoma. *J Clin Oncol* **11**: 1344–1352

Betticher DC, Zucca E, Von Rohr A, Egger T, Radford JA, Ambrosetti A, Bürki K, Rufener B, Hsu Schmitz SF and Cerny T (1996) 2-Chlorodeoxyadenosine (2-CDA) therapy in previously untreated patients with follicular stage III–IV non-Hodgkin's lymphoma. *Ann Oncol* **7**: 793–799

Cabanillas F, Grillo-Lopez AJ, McLaughlin P, Link BK, Levy R, Czuczman M, Heyman MR, Williams M, Jain V, Bence-Bruckler I, Ho AD, Lister J, Rogers J, Shen D and Varns C (1996) Anti-CD20 antibody (MAB), IDEC-C2B8: clearance of bcl-2 t(14;18) positive cells from peripheral blood (PB) and bone marrow (BM) in patients (pts) with relapsed low-grade or follicular lymphoma (LG/F NHL). *Blood* **88** (suppl. 1): 91a

Corbally N, Grogan L, Dervan PA and Carney DN (1992) The detection of specific gene rearrangements in non-Hodgkin's lymphoma using the polymerase chain reaction. *Br J Cancer* **66**: 805–809

Cotter F, Price C, Zucca E and Young BD (1990) Direct sequence analysis of the 14q+ and 18q– chromosome junctions in follicular lymphoma. *Blood* **76**: 131–135

Cotter FE, Price C, Meerabux J, Zucca E and Cavalli F (1991) Direct sequence analysis of $14_{q+}$ and $18_{q-}$ chromosome junctions at MBR and MCR revealing clustering within the MBR in follicular lymphoma. *Ann Oncol* **2** (suppl.2): 93–97

Croce CM (1993) Molecular biology of lymphomas. *Semin Oncol* **20**: 31–46

Dolken G, Illerhaus G, Hirt C and Mertelsmann R (1996) BCL-2/$J_H$ rearrangements in circulating B cells of healthy blood donors and patients with non malignant diseases. *J Clin Oncol* **14**: 1333–1344

Finke J, Slanina J, Lange W and Dolken G (1993) Persistence of circulating t(14;18)-positive cells in long term remission after radiation therapy for localized-stage follicular lymphoma. *J Clin Oncol* **11**: 1668–1673

Gribben JG, Freedman AS, Woo SD, Blake K, Shu RS, Freeman G, Longtine JA, Pinkus GS and Nadler LM (1991) All advanced non-Hodgkin's lymphomas with a polymerase chain reaction amplifiable breakpoint of bcl-2 have residual cells containing the bcl-2 rearrangement at evaluation and after treatment. *Blood* **78**: 3275–3280

Gribben JG, Neuberg D, Barber M, Moore J, Pesek KW, Freedman AS and Nadler LM (1994) Detection of residual lymphoma cells by polymerase chain reaction in peripheral blood is significantly less predictive for relapse than detection in bone marrow. *Blood* **83**: 3800–3807

Hardingham JE, Kotasek D, Sage RE, Gooley LT, Mi JX, Dobrovic A, Norman JE, Bolton AE and Dale BM (1995) Significance of molecular marker-positive cells after autologous peripheral-blood stem-cell transplantation for non-Hodgkin's lymphoma. *J Clin Oncol* **13**: 1073–1079

Harris NL, Jaffe ES, Stein H, Banks PM, Chan JKC, Cleary ML, Delsol G, De Wolf-Peeters C, Falini B, Gatter KC, Grogan TM, Isaacson PG, Knowles DM, Mason DY, Muller-Hermelink HK, Pileri SA, Piris MA, Ralfkiaer E and Warnke RA (1994) A revised European-American classification of lymphoid neoplasms: a proposal from the International Lymphoma Study Group. *Blood* **84**: 1361–1392

Hickish TF, Purvies H, Mansi J, Soukop M and Cunningham D (1991) Molecular monitoring of low grade non-Hodgkin's lymphoma by gene amplification. *Br J Cancer* **64**: 1161–1163

Hockenberry D, Nunez G, Milliman C, Schreiber RD and Korsmeyer SJ (1990) Bcl-2 is an inner mitochondrial membrane protein that blocks programmed cell death. *Nature* **348**: 334–336

Ji W, Qu G, Ye P, Zhang XY, Halabi S and Ehrlich M (1995) Frequent detection of bcl-2/JH translocation in human blood and organ samples by a quantitative polymerase chain reaction assay. *Cancer Res* **55**: 2876–2882

Johnson A, Brun A, Dictor M, Rambech E, Akerman M and Anderson H (1995) Incidence and prognostic significance of t(14;18) translocation in follicle center cell lymphoma of low and high grade. *Ann Oncol* **6**: 789–794

Lambrechts AC, Hupkes PE, Dorssers LCJ and Van't Veer MB (1993) Translocation (14;18)-positive cells are present in the circulation of the majority of patients with localized (stage I and II) follicular non-Hodgkin's lymphoma. *Blood* **82**: 2510–2516

Lambrechts AC, Hupkes PE, Dorssers LCJ and Van't Veer MB (1994) Clinical significance of t(14;18)-positive cells in the circulation of patients with stage III or IV follicular non-Hodgkin's lymphoma during first remission. *J Clin Oncol* **12**: 1541–1546

Limpens J, Stad R, Vos C, De Vlaam C, De Jong D, Van Ommen GJB, Schuuring E and Kluin PM (1995) Lymphoma-associated translocation t(14;18) in blood B cells of normal individuals. *Blood* **85**: 2528–2536

Liu Y, Hernandez AM, Shibata D and Cortopassi GA (1994) BCL2 translocation frequency rises with age in humans. *Proc Natl Acad Sci USA* **91**: 8910–8914

Lopez-Guillermo A, Lee MS, Pugh W, McLaughlin P and Cabanillas F (1996) The molecular breakpoint site of bcl-2 gene has prognostic importance in indolent follicular lymphoma (FL). *Blood* **88** (suppl. 1): 293a

Mrozek K and Bloomfield D (1993) Cytogenetics of indolent lymphomas. *Semin Oncol* **20**: 47–57

Pezzella F, Jones M, Ralfkiaer E, Ersboll J, Gatter KC and Mason DY (1992) Evaluation of *bcl-2* expression and 14;18 translocation as prognostic markers in follicular lymphoma. *Br J Cancer* **65**: 87–89

Price CGA, Meerabux J, Murtagh S, Cotter FE, Rohatiner AZS, Young BD and Lister TA (1991) The significance of circulating cells carrying t(14;18) in long remission from follicular lymphoma. *J Clin Oncol* **9**: 1527–1532

The International Non-Hodgkin's Lymphoma Prognostic Factors Project (1993) A predictive model for aggressive non-Hodgkin's lymphoma. *N Engl J Med* **329**: 987–994

The Non-Hodgkin's Lymphoma Pathologic Classification Project (1982) National Cancer Institute Sponsored Study of classifications of non-Hodgkin's lymphomas. *Cancer* **49**: 2112–2135

Tilly H, Rossi A, Stamatoullas A, Lenormand B, Bigorgne C, Kunlin A, Monconduit M and Bastard C (1994) Prognostic value of chromosomal abnormalities in follicular lymphoma. *Blood* **84**: 1043–1049

Weiss LM, Warnke RA, Sklar J and Cleary ML (1987) Molecular analysis of the t(14;18) chromosomal translocation in malignant lymphomas. *N Engl J Med* **317**: 1185–1189

Whang-Peng J, Knutsen T, Jaffe ES, Raffeld M, Zhao WP, Duffey P, Condron K, Yano T and Longo DL (1995) Sequential analysis of 43 patients with non-Hodgkin's lymphoma: clinical correlation with cytogenetic, histologic, immunophenotyping and molecular studies. *Blood* **85**: 203–216

Yuan R, Dowling P, Zucca E, Diggelman H and Cavalli F (1993) Detection of bcl-2/JH rearrangement in follicular and diffuse lymphoma: concordant results of peripheral blood and bone marrow analysis at diagnosis. *Br J Cancer* **67**: 922–925

Yunis JJ, Frizzera G, Oken MM, McKenna J, Thelogides A and Arnesen M (1987) Multiple recurrent genomic defects in follicular lymphoma. *N Engl J Med* **316**: 79–84

© Cancer Research Campaign 1997